# Anti-Neuronal Autoantibodies (Cell Surface and Onconeural) and Their Association With Natural Autoantibodies in Synthetic Cannabinoid-Induced Psychosis

**DOI:** 10.3389/fpsyt.2022.850955

**Published:** 2022-05-02

**Authors:** Lídia Hau, Tamás Tényi, Natália László, Márton Áron Kovács, Szabina Erdö-Bonyár, Zsuzsanna Csizmadia, Tímea Berki, Diána Simon, Györgyi Csábi

**Affiliations:** ^1^Department of Pediatrics, Clinical Centre, University of Pécs Medical School, Pécs, Hungary; ^2^Department of Psychiatry and Psychotherapy, Clinical Center, University of Pécs Medical School, Pécs, Hungary; ^3^Department of Immunology and Biotechnology, Clinical Center, University of Pécs Medical School, Pécs, Hungary

**Keywords:** autoimmune encephalitis, synthetic cannabinoid, anti-citrate synthase antibodies, psychosis, anti-neuronal autoantibodies, natural autoantibodies

## Abstract

Patients suffering from encephalitis may present psychiatric symptoms; however, the clinical relevance of anti-neuronal antibodies in patients experiencing a psychotic episode without encephalitis is still unclear. In this study, we examined the presence of anti-neuronal cell surface autoantibodies and onconeural autoantibodies in serum samples of 22 synthetic cannabinoid users presenting with psychosis. We found only two positive cases; however, seven patients had borderline results. Nonetheless, we found no significant correlation between anti-neuronal autoantibodies and the intensity of psychosis indicated by the Positive and Negative Syndrome Scale (PANSS) scores. The length of drug use and the combination of other drugs with synthetic cannabinoids have no significant effect on anti-neuronal autoantibody positivity. Nonetheless, the ratio of anti-citrate synthase (anti-CS) IgM and IgG natural autoantibodies was significantly lower (*p* = 0.036) in the anti-neuronal autoantibody-positive/borderline samples, than in the negative group. Interestingly, anti-CS IgM/IgG showed a significant negative correlation with PANSS-positive score (*p* = 0.04, *r* = −0.464). Our results demonstrated that anti-neuronal autoantibody positivity occurs in synthetic cannabinoid users, and the alteration of anti-CS IgM/IgG natural autoantibody levels points to immunological dysfunctions in these cases.

## Introduction

The etiology of psychosis is complex and multifactorial; immunological hypothesis has recently become increasingly prominent in psychiatric research. Multiple studies have identified associations between infections or autoimmune diseases and psychotic disorders (1). The autoimmune neurological disease, N-methyl-D-aspartate (NMDA)-encephalitis, frequently occurs with symptoms characteristic of mental disease (2, 3). A systematic review showed that among patients who are treated with first episode psychosis, anti-neuronal antibodies, including anti-NMDA, were present at a higher rate than in controls (4). Other cell surface autoantibodies, such as voltage-gated potassium channel (VGKC) antibodies, are associated with limbic encephalitis along with insomnia, autonomic dysfunction, neuromyotonia, and cognitive dysfunction in a disease called Morvan syndrome (5). A case report suggests that an onconeural autoantibody, called anti-Yo antibody, might play a role in the induction of psychosis without paraneoplastic neurological syndrome (6). Several other anti-neuronal antibodies have been reported in patients with psychosis or in patients with encephalitis showing psychotic symptoms (7, 8). Therefore, autoantibodies directed against neuronal cell surface or intracellular antigens may have a probable role in mental diseases, especially in psychosis. Nonetheless, the clinical relevance of any of these autoantibodies in patients with psychosis without encephalitis is still not known. Natural antibodies are present in healthy individuals without prior antigenic stimulation, and also in patients with autoimmune diseases (9). Naturally occurring autoantibodies of the IgM isotype are thought to provide protection against autoimmune reactions associated with pathological autoantibodies. The level of natural IgG autoantibodies in sera is higher in patients with various diseases than in healthy individuals and they may represent a breakdown in central tolerance (10).

Synthetic cannabinoid receptor agonists (SCRAs) were synthetized in the 1960s to investigate possible therapeutic effects, and to study cannabinoid receptors (11). In the early 2000s, variations in SCRAs started to sell commercially, by the name “K2, Herbal, Spice, Mojo” and by many other names. They are popular among younger adults and teenagers, because they are cheap, “natural,” and undetectable during routine drug screening. SCRAs mostly have the same effect as tetrahydrocannabinol (THC), which can be found in marihuana. Several studies suggest the potential effect of SCRAs in the treatment of some psychiatric disorders. Medical cannabis and synthetic cannabinoids, both acting on the endocannabinoids system, may have a potential therapeutic use for improving posttraumatic stress disorder (PTSD) and schizophrenia symptoms or inhibit pain (12, 13). Most studies emphasize the immunosuppressive effect of THC and cannabidiol (CBD) (14, 15). Moreover, THC and CBD are currently being investigated as potential therapeutic agents for several inflammatory or autoimmune diseases. However, a few studies suggest their proinflammatory effect in the brain (15). Similar to THC SCRAs bind to cannabinoid receptor 1 (CB1) and to cannabinoid receptor 2 (CB2) and stimulate CB1 more than CB2. CB1 are found in the central nervous system, especially in the cerebral cortex, hippocampus, cerebellum, and basal ganglia. CB2 are mostly expressed by immune (macrophage and B cells) and hematopoietic cells; thus, stimulation of CB2 has immunomodulatory effects (11, 16). CB2 seems to play an important role in the immune mechanism in the central nervous system (17). Several case reports suggest that cannabis can cause vasculitis both in peripheral arteries and in the central nervous system by autoimmune reactions (18–20). THC exposure during adolescence also resulted in a persistent neuroinflammatory state in adult female rats and mice, characterized by altered microglia morphologic structure, increased proinflammatory mediators, reduced CB1, and increased CB2 (21). A few case reports show that SCRA users can develop autoimmune disease (22, 23). The study by Parajuli et al. represents a case about a drug-induced posterior reversible encephalopathy syndrome (PRES) after K2 consumption (a type of SCRAs); besides, the autoimmune mechanism of a toxic origin can be found in the background of PRES (24). Furthermore, in our previous study, we reported the case of a teenager who used SCRA and was diagnosed with NMDA encephalitis (16). However, the relationship between the use of SCRAs and the presence of anti-neuronal antibodies was not investigated in detail. Consequently, the main purpose of this study was to find correlations between anti-neuronal antibodies and the intensity of psychosis indicated with Positive and Negative Syndrome Scale (PANSS) score in SCRA users. Further aim was to search for possible associations between natural autoantibodies and anti-neuronal autoantibodies with possible relevance to the assessment of the severity of drug-induced psychosis.

## Methods

### Patients

The study is based on the data of 22 patients with suspected SCRAs-induced psychosis (Table 1). All the patients underwent a comprehensive psychiatric evaluation and assessment of acute psychotic exacerbation of PANSS. Inclusion criteria were as follows: psychosis after using SCRAs, adolescents and young adults (age between 13 and 32 years), normal serum electrolytes, blood counts, kidney, and liver function, and signed written informed consent form. General exclusion criteria were the diagnosis of schizophrenia, schizoaffective psychosis, bipolar disorder, autoimmune disorders, and ongoing infection. The study was approved by the Regional Clinical Research Committee (5951-PTE2015). Peripheral blood was collected and allowed to clot for at least 30 min before centrifugation for 10 min at 1,000 × *g*. Serum was removed and stored at −80°C until performing the tests for determination of autoantibodies.

**Table 1:** Characteristics of patients.

**Characteristics**	**Synthetic cannabinoid users (*n* = 22)**
Age (years), mean (SD)	17 (4.9)
Sex (male), *n* (%)	19 (86.4%)
Family history (positive for addiction), *n* (%)	5 (22.7%)
Polytoxicomania (yes), *n* (%)	9 (40.9%)
Drug use (month), mean (SD)	23.8 (23.5)
PANSS total, mean (SD)	55 (18)
PANSS general, mean (SD)	32.6 (8.7)
PANSS positive, mean (SD)	11.7 (7.6)
PANSS negative, mean (SD)	11.1 (5.5)

*PANSS, Positive and Negative Syndrome Scale*.

### Detection of Anti-Neuronal Autoantibodies

The anti-neuronal autoantibodies were detected either with indirect immunofluorescence or immunoblot techniques. IgG antibodies directed against neuronal cell surface antigens, including N-methyl-D-aspartate-type glutamate receptor (NMDA), alpha-amino-3-hydroxy-5-methyl-4-isoxazolepropionic acid receptor (AMPA1, AMPA2), contactin-associated protein 2 (CASPR2), leucine-rich glioma-inactivated protein 1 (LGI1), and gamma- aminobutyric acid beta receptor (GABA B receptor), were detected simultaneously using a biochip mosaic of transfected HEK293 cells expressing these six antigens of interest (Autoimmune Encephalitis Mosaic 1; Euroimmun, Lübeck, Germany). Samples were classified as positive, borderline, or negative based on the fluorescence intensity of the transfected cells. Onconeural antibodies (IgG antibodies targeting intracellular antigens), namely, glutamic acid decarboxylase-65 (GAD65), collapsin response mediator protein 5/crossveinless-2 (CV2), type 1 anti-neuronal nuclear antibody (ANNA-1, Hu), Ri, Yo, Ma2/Ta, zinc finger protein 4 (ZIC4), amphiphysin (Amp), recovering (Rec), titin, sry-like high mobility groupbox protein1 (SOX1), and Tr/delta notch-like epidermal growth factor-related receptor (DNER), were determined using EUROLINE paraneoplastic neurologic syndromes 12 Ag test (Euroimmun, Lübeck, Germany). For the evaluation of the test strips, the recommended EUROLineScan software was used, which automatically identifies the bands on the test strip and measures their intensity. Based on the intensity of the bands, the result of the autoantibody test can be negative, borderline, or positive.

### Measurement of Natural Autoantibodies

We have previously shown that antibodies directed against citrate synthase belong to the pool of natural autoantibodies (9). The levels of anti-citrate synthase (anti-CS) IgM and IgG autoantibodies were determined with an in-house ELISA, as previously described (9). Briefly, 96-well polystyrene plates were coated with 100 μl of 5 μg/ml citrate synthase from porcine heart (Sigma, St Louis, MO, USA) at 4–8°C overnight. Following the saturation of nonspecific binding sites, serum samples were incubated in duplicate at 1:100 dilution for 1 h at room temperature. Finally, the plate was incubated with horseradish peroxidase (HRP)-conjugated anti-human IgM or IgG-specific antibodies (Dako, Glostrup, Denmark) for 1 h at room temperature; the reaction was developed with TMB and measured at 450 nm, using an iEMS MF microphotometer (Thermo Labsystem, Beverly MA, USA).

### Statistical Analysis

Statistical evaluation was performed with SPSS v. 27.0 statistics package (IBM, Armonk, NY, USA). Continuous variables were compared with the Mann-Whitney *U* test; Fischer's exact test was used to find the difference between categorical variables. Relationship between continuous variables was assessed with Spearman correlation. A *p* < 0.05 was considered significant.

## Results

Samples of eight of the 22 patients (36.4%) had positive or borderline results for anti-neuronal autoantibodies. One patient (4.5%) was positive for the antibody against CASPR2 from the neuronal cell surface antigens. One patient (4.5%) showed anti-AMP positivity, and six patients (27.3%) had a borderline result for other onconeural antibodies targeting Rec, Yo, Hu, SOX1, and Tr. None of these patients received a diagnosis of autoimmune encephalitis (Table 2).

**Table 2:** Clinical features and autoantibody results of each case.

**Age** **and sex**	**Previous** **history of** **psychiatric** **disorders**	**Duration of** **drug use** **(in months)**	**Substances**	**Cell surface antigens**	**Onconeural antigens**	**PANSS positive**	**PANSS negative**	**PANSS general**	**PANSS** **total**
15, male	–	48	SCRA	negative	Yo borderline	9	10	22	41
16, male	1	6	SCRA	negative	Rec borderline	7	7	25	39
17, male	–	9	SCRA	negative	negative	7	27	46	80
16, male	–	3	SCRA	negative	Hu borderline	7	7	32	46
15, male	–	–	SCRA, other NPS	negative	Tr borderline	26	13	54	93
17, male	–	–	SCRA	negative	SOX1 borderline	7	7	31	45
15, male	–	3	SCRA	negative	negative	10	7	34	51
13, female	2	<1	SCRA	negative	negative	7	11	32	50
17, female	3	60	SCRA	negative	negative	7	7	26	40
16, male	3	48	SCRA, NC, LSD, other NPS, MDMA, amphetamine	negative	Amp positive	7	14	35	56
17, male	3	3	SCRA, NC	negative	negative	7	7	34	48
15, female	4	4	SCRA	negative	negative	7	7	22	36
17, male	–	<1	SCRA	negative	negative	7	10	32	49
15, male	3	24	SCRA	negative	negative	7	7	28	42
13, male	1	18	SCRA	negative	negative	7	7	32	46
14, male	5	60	SCRA, cocaine, other NPS	negative	Rec borderline	7	13	27	47
17, male	–	60	SCRA, amphetamine, NC	negative	negative	23	8	32	63
15, male	4	36	SCRA, NC, MDMA, cocaine	negative	negative	7	13	30	50
17, male	–	60	SCRA	negative	negative	23	16	46	85
19, male	–	8	SCRA, BDZ	negative	negative	24	19	41	84
32, male	6	17	SCRA, other NPS	negative	negative	28	21	39	88
30, male	7	6	SCRA, cocaine, heroin, NC	CASPR2 positive	negative	16	7	17	40
**Previous history of psychiatric disorders:**									
1. Attention deficit hyperactivity disorder									
2. Emotional disorders with onset specific to childhood									
3. Unspecified behavioral and emotional disorders with onset usually occurring in childhood and adolescence									
4. Adjustment disorders									
5. Mild mental retardation									
6. Personality disorder, unspecified									
7. Other acute and transient psychotic disorders									

*SCRA, Synthetic Cannabinoid Receptor Agonist; NPS, New Psychoactive Substances; NC, Natural Cannabis; MDMA, 3,4-methylenedioxymethamphetamine; LSD, Lysergic Acid Diethylamide; BDZ, Benzodiazepine*.

The clinical background of two interesting adolescent patients is presented, one with long-term drug use (for 4 years) and another with short-term drug use (for 6 months) (Table 3). Both patients are anti-neuronal antibody-positive or borderline cases.

**Table 3:** Case vignettes for two patients with positive or borderline anti-neuronal antibody test result.

**Case vignette 1**
Case 1 was a 16-year-old adolescent who had been using drugs for 4 years; previously, he had consumed different drugs. In the past period, he used synthetic cannabinoids; the last time when he used it was on the same day of admittance to hospital. His serum sample was positive for anti-AMP antibody. After his admission, he tried to escape many a times, and he was aggressive with the nurses. He had psychomotor agitation, acoustic hallucination, and had suicidal intention after using synthetic cannabinoids. He tried to stop using drugs but failed.
**Case vignette 2**
Case 2 was a 16-year-old adolescent who had been using drugs for 6 months. He was adopted and started using synthetic cannabinoids when his foster mother died. He admitted not using drugs for 2 weeks. Anti-Rec antibody was borderline in his laboratory findings. He behaved aggressively due to substance use in the past months. He had burst of anger many a times against his mates, and he damaged the furniture in the orphanage. Once he threatened his classmate with an alarm gun.

For statistical analyses, patients with positive and borderline results for anti-neuronal autoantibodies were considered as one group. We found no significant difference in PANSS-total, PANSS-positive, PANSS-negative, and PANSS-general scores between patients with positive/borderline and negative results. The length of drug use and the combination of other drugs with synthetic cannabinoids had no significant effect on anti-neuronal autoantibody positivity.

**Figure 1:**
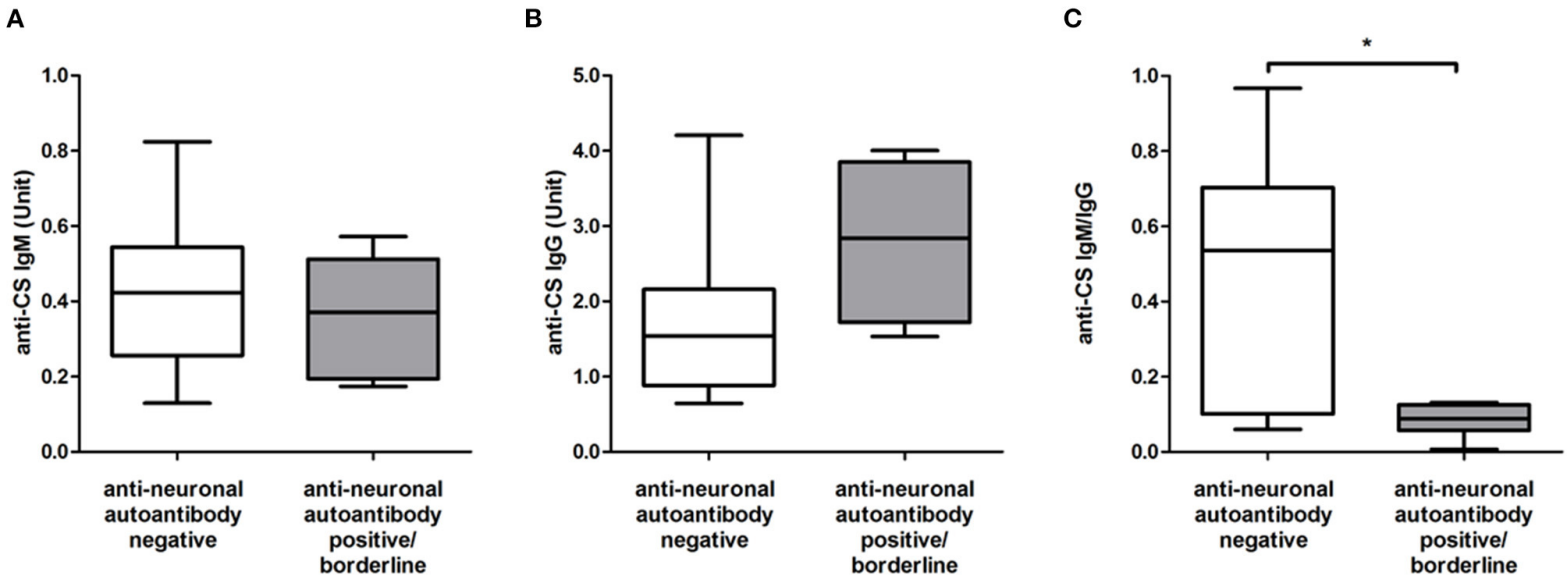
The level of anti-citrate synthase (CS) IgM **(A)**, IgG **(B)**, and autoantibodies and their ratio **(C)** in patients with positive/borderline (*n* = 8) and negative (*n* = 14) results for anti-neuronal autoantibodies. The boxes show interquartile ranges (IQR); the horizontal lines represent medians and the whiskers indicate the lowest and highest values. *p < 0.05.

We also looked for alterations in the level of natural anti-CS IgM and IgG autoantibodies between patients with anti-neuronal autoantibody-positive/borderline and negative results. We found no significant differences neither in the level of anti-CS IgM (Figure 1A) nor in the level of anti-CS IgG antibodies (Figure 1B) between the anti-neuronal autoantibody-positive/borderline and the negative group; however, the level of anti-CS IgG showed a higher trend in patients with anti-neuronal autoantibody-positive/borderline results than in patients with negative results (Figure 1B). Therefore, we also analyzed the ratio of anti-CS IgM and IgG between these patient groups and found that it was significantly lower (*p* = 0.036) in the anti-neuronal autoantibody-positive/borderline group than in the negative group (Figure 1C). Next, we evaluated the possible associations between the number of anti-CS IgM, IgG antibodies, their ratio, and the severity of symptoms measured by PANSS-total, PANSS-positive, PANSS-negative, and PANSS-general scores. Interestingly, the ratio of anti-CS IgM and IgG showed a significant negative correlation with PANSS-positive score (*p* = 0.04, *r* = −0.464).

## Discussion

The usage of recent designer drugs, including substituted cathinones (mephedrone, methylone, often called as “bath salts”), SCRAs and synthetic hallucinogens (N-bomb) expanded in the past decade, and they are well-known in the market, especially among the young population. Compound availability has changed rapidly, and it is hard to detect these substances on the routine urine drug test (25). The purchase via internet is cheap, as “legal high” promotes widespread use among adolescents. SCRA users are usually poorly educated and mostly males (25–27); in agreement with this, 86.4% of the patients in our study were males. Besides the stimulatory effect of these drugs, acute toxicity and psychosis may occur. Some toxicology reports highlighted the main presenting features being toxic psychosis and delirium (40%), agitation (10%), and hallucinations (4–7%) (25, 26, 28). SCRA users had higher levels of positive PANSS than THC users. Greater toxicity can be attributed to pharmacological features: SCRAs show 50–300 times greater affinity for the CB1 than THC and they are full agonists at CB1 (27). A report suggests that CB1 antagonist rimonabant could be a treatment option for the management of SCRA overdose (29). Rimonabant was used as an antiobesity drug, but it was withdrawn in Europe because of psychiatric side effects in 2008. Additional studies are required to apply for its possible application in other medical conditions. Cannabinoids can modulate immune reactions in the brain (17), and in our study, we found that 36.4% of the patients with SCRA-induced psychosis had a positive or borderline result for anti-neuronal autoantibodies. Among the autoantibodies against neuronal cell surface antigens, the one most commonly investigated is the anti-NMDA antibody. A recent study found anti-NMDA IgG in 8.6% patients suffering from schizophrenia, but interestingly, healthy controls showed an even higher rate (10.8%) of positivity (30, 31). However, none of the patients in our study had anti-NMDA antibody. LGI1 and CASPR2 antibodies are currently classified as VGKC complex antibodies and are commonly considered to have the same clinical significance (32). There are cases where anti-VGKC complex disease initially presented with schizophreniform psychiatric disease (33, 34). In our study, only one patient had a borderline result for the antibody against CASPR2. Onconeural antibodies were suggested to contribute to immunological alterations in patients with psychiatric disorders, but the literature on these antibodies in psychiatric diseases is scarce (21). Anti-Hu and anti-Yo antibodies were shown to induce neuronal and Purkinje-cell death in the hippocampal and cerebellar regions of rats (35–37). Case reports suggested that anti-Yo and anti-Ri onconeural antibodies may play a role in autoimmune processes in patients with psychiatric disease (6, 38, 39). Only one of our patients was positive for anti-Amp antibody, two patients had borderline result for anti-Rec, and four patients showed a borderline result for anti-Yo, anti-Hu, anti-SOX1, or anti-Tr antibodies. We did not find any significant correlations between anti-neuronal antibodies and the PANSS scores of the investigated patients, suggesting that anti-neuronal antibodies do not influence the severity of SCRA-induced psychosis. Neuroscientific studies have identified atypical dopamine activity in cannabis users, which therefore could underlie its association with psychosis in SCRA users (40). In our previous studies, we detected natural autoantibodies directed against CS in healthy individuals and patients with autoimmune diseases (9, 41, 42). Natural IgM autoantibodies are polyreactive; they recognize evolutionally conserved self-structures and serve as scavengers of damaged molecules and cells. They participate in the removal of apoptotic cells and maintain tissue homeostasis, and therefore, have been implicated in the control of inflammation and immunological balance (43–45). The majority of natural autoantibodies was originally thought to be of IgM isotype, but later, the presence of natural IgG autoantibodies was also described and their presence could be the result of an adaptive-like immune response (9, 42). Under pathological conditions, a compensatory increase in IgG antibodies with anti-idiotypic activity can occur (46), and previously, we found an elevated level of anti-CS IgG antibodies in patients with systemic lupus erythematosus positive for anti-dsDNA IgG (41). Consequently, the higher trend in anti-CS IgG level, which resulted in an decreased ratio of anti-CS IgM/IgG autoantibodies in patients with anti-neuronal autoantibody-positive/borderline results, may be a harbinger of autoimmune phenomena.

We can conclude that the presence of anti-neuronal autoantibodies in serum samples of patients acutely admitted to hospital with a psychotic episode induced by SCRAs abuse is not exceptional; however, routine screening for these antibodies is not likely to be informative in most cases. According to our results, testing for anti-neuronal antibodies in serum cannot be suggested for diagnostic purposes in patients using SCRA, as their detection has no therapeutic impact on these cases. Additional studies are required to check the presence of these antibodies in the cerebrospinal fluid (CSF). To our knowledge, this is the first study addressing the prevalence of anti-neuronal and natural autoantibodies among SCRA users. Our study has the limitation that healthy or other psychotic adolescent controls were not enrolled, but for a pediatric patient group, it is hard to find age-matched healthy volunteers with the permission of parents. Nevertheless, our aim was to investigate the relevance of anti-neuronal autoantibody positivity in SCRA users. Other limitation of this study was the negative result of urine and serum tests for synthetic cannabinoids. The clinical challenge of these substances is that the chemical variety makes monitoring difficult.

## Data Availability Statement

The original contributions presented in the study are included in the article/supplementary material, further inquiries can be directed to the corresponding author.

## Ethics Statement

The studies involving human participants were reviewed and approved by Regional Clinical Research Committee (5951-PTE2015). Written informed consent to participate in this study was provided by the participants' legal guardian/next of kin. Written informed consent was obtained from the individual(s), and minor(s)' legal guardian/next of kin, for the publication of any potentially identifiable images or data included in this article.

## Author Contributions

TT and DS designed the study. LH, GC, TT, SE-B, ZC, and DS performed the experiments. LH, GC, NL, MK, and TT contributed to the clinical data. LH, TB, and DS analyzed the data. LH and DS wrote the first draft of the manuscript. All authors contributed to the article and approved the submitted version of the manuscript.

## Funding

This study was supported by the János Bolyai Research Scholarship of the Hungarian Academy of Sciences, Hungarian National Brain Research Programme KTIA-13-NAP-A-II/12(2018-2022), ÚNKP-21-5 New National Excellence Program of the Ministry for Innovation and Technology from the source of the National Research, Development and Innovation Fund, and the National Excellence Program (FIKP-II) and by the Thematic Excellence Program 2020 (TKP2020)–Institutional Excellence Sub-program of the Ministry for Innovation and Technology in Hungary, within the framework of the second thematic program of the University of Pécs.

## Conflict of Interest

The authors declare that the research was conducted in the absence of any commercial or financial relationships that could be construed as a potential conflict of interest.

## Publisher's Note

All claims expressed in this article are solely those of the authors and do not necessarily represent those of their affiliated organizations, or those of the publisher, the editors and the reviewers. Any product that may be evaluated in this article, or claim that may be made by its manufacturer, is not guaranteed or endorsed by the publisher.

## References

[1] Runge K Fiebich BL Kuzior H Saliba SW Yousif NM Meixensberger S. An observational study investigating cytokine levels in the cerebrospinal fluid of patients with schizophrenia spectrum disorders. Schizophr Res. (2021) 231:205–13. 10.1016/j.schres.2021.03.022 33887648

[2] Hau L Csábi G Tényi T. Anti-N-methyl-D aspartate receptor encephalitis - guideline to the challenges of diagnosis and therapy. Psychiatr Hung. (2015) 30:402–8. 26771699

[3] Pollak TA Beck K Irani SR Howes OD David AS McGuire PK. Autoantibodies to central nervous system neuronal surface antigens: psychiatric symptoms and psychopharmacological implications. Psychopharmacology (Berl). (2016) 233:1605–21. 10.1007/s00213-015-4156-y 26667479 PMC4828500

[4] Ezeoke A Mellor A Buckley P Miller B. A systematic, quantitative review of blood autoantibodies in schizophrenia. Schizophr Res. (2013) 150:245–51. 10.1016/j.schres.2013.07.029 23953827

[5] Buckley C Oger J Clover L Tüzün E Carpenter K Jackson M. Potassium channel antibodies in two patients with reversible limbic encephalitis. Ann Neurol. (2001) 50:73–8. 10.1002/ana.1097 11456313

[6] Endres D Perlov E Stich O Meyer PT Lützen N Tebartz van Elst L. Case report: low-titre anti-Yo reactivity in a female patient with psychotic syndrome and frontoparieto-cerebellar atrophy. BMC Psychiatry. (2015) 15:112. 10.1186/s12888-015-0486-x 25963777 PMC4436095

[7] Leypoldt F Armangue T Dalmau J. Autoimmune encephalopathies. Ann N Y Acad Sci. (2015) 1338:94–114. 10.1111/nyas.12553 25315420 PMC4363225

[8] Mantere O Saarela M Kieseppä T Raij T Mäntylä T Lindgren M. Anti-neuronal anti-bodies in patients with early psychosis. Schizophr Res. (2018) 192:404–7. 10.1016/j.schres.2017.04.027 28461116

[9] Czömpöly T Olasz K Simon D Nyárády Z Pálinkás L Czirják L. A possible new bridge between innate and adaptive immunity: Are the anti-mitochondrial citrate synthase autoantibodies components of the natural antibody network? Mol Immunol. (2006) 43:1761–8. 10.1016/j.molimm.2005.11.004 16368144

[10] Nagele EP Han M Acharya NK DeMarshall C Kosciuk MC Nagele RG. Natural IgG autoantibodies are abundant and ubiquitous in human sera, and their number is influenced by age, gender, and disease. PLoS ONE. (2013) 8:e60726. 10.1371/journal.pone.0060726 23589757 PMC3617628

[11] Mills B Yepes A Nugent K. Synthetic Cannabinoids. Am J Med Sci. (2015) 350:59–62. 10.1097/MAJ.0000000000000466 26132518

[12] Orsolini L Chiappini S Volpe U Berardis D Latini R Papanti GD. Use of medicinal cannabis and synthetic cannabinoids in post-traumatic stress disorder (PTSD): a systematic review. Medicina (Kaunas). (2019) 55. 10.3390/medicina55090525 31450833 PMC6780141

[13] Gambi F De Berardis D Sepede G Quartesan R Calcagni E Salerno RM. Cannabinoid receptors and their relationships with neuropsychiatric disorders. Int J Immunopathol Pharmacol. (2005) 18:15–9. 10.1177/039463200501800103 15698507

[14] Giorgi V Marotto D Batticciotto A Atzeni F Bongiovanni S Sarzi-Puttini P. Cannabis and autoimmunity: possible mechanisms of action. Immunotargets Ther. (2021) 10:261–71. 10.2147/ITT.S267905 34322454 PMC8313508

[15] Da Silva T Hafizi S Watts JJ Weickert CS Meyer JH Houle S. In vivo imaging of translocator protein in long-term cannabis users. JAMA Psychiatry. (2019) 76:1305–13. 10.1001/jamapsychiatry.2019.2516 31532458 PMC6751758

[16] Hau L Csabi G Rozsai B Stankovics J Tenyi T Hollody K. Anti-N-methyl-D-aspartate receptor encephalitis and drug abuse - the probable role of molecular mimicry or the overstimulation of CB receptors in a 17-year-old adolescent - case report. Neuropsychopharmacol Hung. (2016) 18:162–4. 27824312

[17] Tanasescu R Constantinescu CS. Cannabinoids and the immune system: an overview. Immunobiology. (2010) 215:588–97. 10.1016/j.imbio.2009.12.005 20153077

[18] Barrio P Balcells M La Puma D Gaig C. Autoimmune-mediated psychosis: a case of susac syndrome in a drug user. J Dual Diagn. (2017) 13:133–5. 10.1080/15504263.2017.1296212 28368694

[19] Dhadwal G Kirchhof MG. The risks and benefits of cannabis in the dermatology clinic. J Cutan Med Surg. (2018) 22:194–9. 10.1177/1203475417738971 29056081

[20] El Omri N Eljaoudi R Mekouar F Jira M Sekkach Y Amezyane T. Cannabis arteritis. Pan Afr Med J. (2017) 26:53. 10.11604/pamj.2017.26.178.11763 28451030 PMC5398854

[21] Zamberletti E Gabaglio M Prini P Rubino T Parolaro D. Cortical neuroinflammation contributes to long-term cognitive dysfunctions following adolescent delta-9-tetrahydrocannabinol treatment in female rats. Eur Neuropsychopharmacol. (2015) 25:2404–15. 10.1016/j.euroneuro.2015.09.021 26499171

[22] Castaneira G Rojas K Galili Y Field Z Perez-Perez A Madruga M. Idiopathic thrombocytopenic purpura induced by synthetic cannabinoid. J Addict Med. (2019) 13:235–6. 10.1097/ADM.0000000000000485 30531235

[23] Sinangil A Celik V Kockar A Ecder T. synthetic cannabinoid induced acute tubulointerstitial nephritis and uveitis syndrome: a case report and review of literature. J Clin Diagn Res. (2016) 10:OD31–2. 10.7860/JCDR/2016/18762.7826 27437289 PMC4948465

[24] Parajuli P Regmi MR Lara-Garcia OE Abu Limon I Deckard A. Man vs. man-made marijuana: A case of drug-induced posterior reversible encephalopathy syndrome (PRES) due to K2, a ynthetic cannabinoid (SCB). J Community Hosp Intern Med Perspect. (2020) 10:361–4. 10.1080/20009666.2020.1781349 32850099 PMC7427457

[25] Weaver MF Hopper JA Gunderson EW. Designer drugs 2015: assessment and management. Addict Sci Clin Pract. (2015) 10:8. 10.1186/s13722-015-0024-7 25928069 PMC4422150

[26] Hobbs M Kalk NJ Morrison PD Stone JM. Spicing it up - synthetic cannabinoid receptor agonists and psychosis - a systematic review. Eur Neuropsychopharmacol. (2018) 28:1289–304. 10.1016/j.euroneuro.2018.10.004 30454908

[27] Kalk NJ Boyd A Strang J Finch E. Spice and all things nasty: the challenge of synthetic cannabinoids. BMJ. (2016) 355:i5639. 10.1136/bmj.i5639 27777237

[28] Loeffler G Hurst D Penn A Yung K. Spice, bath salts, and the U. S military: the emergence of synthetic cannabinoid receptor agonists and cathinones in the US Armed Forces. Mil Med. (2012) 177:1041–8. 10.7205/MILMED-D-12-00180 23025133

[29] Ford BM Tai S Fantegrossi WE Prather PL. Synthetic pot: not your grandfather's marijuana. Trends Pharmacol Sci. (2017) 38:257–76. 10.1016/j.tips.2016.12.003 28162792 PMC5329767

[30] Hammer C Stepniak B Schneider A Papiol S Tantra M Begemann M. Neuropsychiatric disease relevance of circulating anti-NMDA receptor autoantibodies depends on blood-brain barrier integrity. Mol Psychiatry. (2014) 19:1143–9. 10.1038/mp.2013.110 23999527

[31] Endres D Perlov E Baumgartner A Hottenrott T Dersch R Stich O. Immunological findings in psychotic syndromes: a tertiary care hospital's CSF sample of 180 patients. Front Hum Neurosci. (2015) 9:476. 10.3389/fnhum.2015.00476 26441585 PMC4564575

[32] van Sonderen A Petit-Pedrol M Dalmau J Titulaer MJ. The value of LGI1, Caspr2 and voltage-gated potassium channel antibodies in encephalitis. Nat Rev Neurol. (2017) 13:290–301. 10.1038/nrneurol.2017.43 28418022

[33] Gnanavel S. Voltage-gated potassium channel (VGKC) antibody-associated encephalopathy presenting as psychosis: a case report. J Neuropsychiatry Clin Neurosci. (2014) 26:E34–5. 10.1176/appi.neuropsych.13070157 25093780

[34] van Elst LT Klöppel S Rauer S. Voltage-gated potassium channel/LGI1 antibody-associated encephalopathy may cause brief psychotic disorder. J Clin Psychiatry. (2011) 72:722–3. 10.4088/JCP.10l06510 21658353

[35] Greenlee JE Clawson SA Hill KE Wood BL Tsunoda I Carlson NG. Purkinje cell death after uptake of anti-Yo antibodies in cerebellar slice cultures. J Neuropathol Exp Neurol. (2010) 69:997–1007. 10.1097/NEN.0b013e3181f0c82b 20838245 PMC2959164

[36] Greenlee JE Clawson SA Hill KE Wood B Clardy SL Tsunoda I. Neuronal uptake of anti-Hu antibody, but not anti-Ri antibody, leads to cell death in brain slice cultures. J Neuroinflammation. (2014) 11:160. 10.1186/s12974-014-0160-0 25228406 PMC4174281

[37] Greenlee JE Clawson SA Hill KE Wood B Clardy SL Tsunoda I. Anti-Yo antibody uptake and interaction with its intracellular target antigen causes Purkinje cell death in rat cerebellar slice cultures: a possible mechanism for paraneoplastic cerebellar degeneration in humans with gynecological or breast cancers. PLoS ONE. (2015) 10:e0123446. 10.1371/journal.pone.0123446 25885452 PMC4401511

[38] Laadhar L Sidhom O Zitouni M Sassi N Abdelghaffar W Lahmar H. High prevalence of antineuronal antibodies in Tunisian psychiatric inpatients. J Neuropsychiatry Clin Neurosci. (2015) 27:54–8. 10.1176/appi.neuropsych.13070153 25111364

[39] Sæther SG Schou M Kondziella D. What is the significance of onconeural antibodies for psychiatric symptomatology? A systematic review. BMC Psychiatry. (2017) 17:161. 10.1186/s12888-017-1325-z 28468645 PMC5415831

[40] Fergusson DM Poulton R Smith PF Boden JM. Cannabis and psychosis. BMJ. (2006) 332:172–5. 10.1136/bmj.332.7534.172 16424500 PMC1336774

[41] Böröcz K Simon D Erdo-Bonyár S Kovács KT Tuba É Czirják L. Relationship between natural and infection-induced antibodies in systemic autoimmune diseases (SAD): SLE, SSc and RA. Clin Exp Immunol. (2021) 203:32–40. 10.1111/cei.13521 32959462 PMC7744489

[42] Czömpöly T Olasz K Nyárády Z Simon D Bovári J Németh P. Detailed analyses of antibodies recognizing mitochondrial antigens suggest similar or identical mechanism for production of natural antibodies and natural autoantibodies. Autoimmun Rev. (2008) 7:463–7. 10.1016/j.autrev.2008.03.006 18558363

[43] Ehrenstein MR Notley CA. The importance of natural IgM: scavenger, protector and regulator. Nat Rev Immunol. (2010) 10:778–86. 10.1038/nri2849 20948548

[44] Grönwall C Vas J Silverman GJ. Protective roles of natural IgM antibodies. Front Immunol. (2012) 3:66. 10.3389/fimmu.2012.00066 22566947 PMC3341951

[45] Grönwall C Silverman GJ. Natural IgM: beneficial autoantibodies for the control of inflammatory and autoimmune disease. J Clin Immunol. (2014) 34 Suppl 1:S12–21. 10.1007/s10875-014-0025-4 24691998 PMC4354681

[46] Abdou NI Wall H Lindsley HB Halsey JF Suzuki T. Network theory in autoimmunity. In vitro suppression of serum anti-DNA antibody binding to DNA by anti-idiotypic antibody in systemic lupus erythematosus. J Clin Invest. (1981) 67:1297–304. 10.1172/JCI110158 6971876 PMC370696

